# Singular and Combined Effects of Essential Oil and Honey of *Eucalyptus Globulus* on Anti-Inflammatory, Antioxidant, Dermatoprotective, and Antimicrobial Properties: In Vitro and In Vivo Findings

**DOI:** 10.3390/molecules27165121

**Published:** 2022-08-11

**Authors:** Hamza M. Assaggaf, Hanae Naceiri Mrabti, Bodour S. Rajab, Ammar A. Attar, Munerah Hamed, Ryan A. Sheikh, Nasreddine El Omari, Naoual El Menyiy, Omar Belmehdi, Shafi Mahmud, Mohammed Merae Alshahrani, Moon Nyeo Park, Bonglee Kim, Gokhan Zengin, Abdelhakim Bouyahya

**Affiliations:** 1 Department of Laboratory Medicine, Faculty of Applied Medical Sciences, Umm Al-Qura University, Makkah 21955, Saudi Arabia; 2 Laboratory of Pharmacology and Toxicology, Bio Pharmaceutical and Toxicological Analysis Research Team, Faculty of Medicine and Pharmacy, Mohammed V University in Rabat, Rabat BP 6203, Morocco; 3 Department of Pathology, Faculty of Medicine, Umm Al-Qura University, Makkah 21955, Saudi Arabia; 4 Biochemistry Department, Faculty of Science, King Abdulaziz University, Jeddah 21589, Saudi Arabia; 5 Laboratory of Histology, Embryology, and Cytogenetic, Faculty of Medicine and Pharmacy, Mohammed V University in Rabat, Rabat BP 6203, Morocco; 6 Laboratory of Pharmacology, National Agency of Medicinal and Aromatic Plants, Taouanate 34025, Morocco; 7 Biology and Health Laboratory, Department of Biology, Faculty of Science, Abdelmalek-Essaadi University, Tetouan 93000, Morocco; 8 Division of Cancer and Genome Sciences, John Curtin School of Medical Research, Australian National University, Canberra, ACT 2601, Australia; 9 Department of Clinical Laboratory Sciences, Faculty of Applied Medical Sciences, Najran University, 1988, Najran 61441, Saudi Arabia; 10 Department of Pathology, College of Korean Medicine, Kyung Hee University, Seoul 02447, Korea; 11 Department of Biology, Science Faculty, Selcuk University, Konya 42130, Turkey; 12 Laboratory of Human Pathologies Biology, Department of Biology, Faculty of Sciences, and Genomic Center of Human Pathologies, Faculty of Medicine and Pharmacy, Mohammed V University in Rabat, Rabat BP 6203, Morocco

**Keywords:** *Eucalyptus globulus*, honey, essential oils, anti-inflammatory, antimicrobial

## Abstract

*Eucalyptus globulus* is a plant widely used by the world population, including Morocco, in the treatment of several pathologies. The aim of this work is to evaluate the antioxidant, anti-inflammatory, dermatoprotective, and antimicrobial effects of essential oil and honey from *E. globulus*, as well as their combination. Chemical composition was determined by GC-MS analysis. The antioxidant activity was evaluated by three tests, namely, DPPH, reducing power, and the β-carotene/linoleic acid assay. The anti-inflammatory activity was investigated in vitro (5-lipoxygenase inhibition) and in vivo (carrageenan-induced paw edema model), while the dermatoprotective activity was tested in vitro (tyrosinase inhibition). Moreover, the antibacterial activity was assessed using agar well diffusion and microdilution methods. The results showed that eucalyptol presents the main compound of the essential oil of *E. globulus* (90.14%). The mixture of essential oil with honey showed the best antioxidant effects for all the tests used (0.07 < IC_50_ < 0.19 mg/mL), while the essential oil was the most active against tyrosinase (IC_50_ = 38.21 ± 0.13 μg/mL) and 5-lipoxygenase (IC_50_ = 0.88 ± 0.01 μg/mL), which corroborated the in vivo test. Additionally, the essential oil showed the best bactericidal effects against all strains tested, with inhibition diameter values ranging from 12.8 to 21.6 mm. The findings of this work showed that the combination of the essential oil with honey showed important results in terms of biological activity, but the determination of the underlying mechanisms of action remains a major prospect to be determined.

## 1. Introduction

Different recent investigations currently support the use of natural resources to identify and develop bioactive compounds. The screening of the biological effects of natural substances passes through several stages, which begin with the identification of the natural source, then the extraction and the identification of the principles, and finishing with biological tests [1,2,3]. The most used natural sources for identifying and isolating active principles include medicinal plants, which are used in an empiric way for the treatment of pathologies. Medicinal plants have the property of synthesizing so-called secondary metabolites [4,5,6].

Natural substances, in particular, essential oils (EOs), are a promising source of biologically active substances because they are characterized by important biological and pharmacological properties, such as antidiabetic, anti-inflammatory, antimicrobial, and anticancer activities [7,8,9,10,11]. These EOs are mainly contained in medicinal and aromatic plants and play some important physiological role in the life of plants. In addition, honey, a natural bee product, is another important source of biologically active molecules originating from medicinal plants. Indeed, we can distinguish, in a conditioned way, the honey characterizing the plant on which the bee feeds, such as the honey of *Eucalyptus*, *Thymus*, and *Origanum*.

Recent studies have shown that honey contains several bioactive molecules, in particular, flavonoids and phenolic acids, and has enormous biological and pharmacological properties. Since honey is a food product, its biological and pharmacological properties constitute a preventive treatment for multiple pathologies [12,13]. On the other hand, the combination of essential oil (inedible and poorly soluble molecules) with honey could facilitate the solubility of essential oils and also their intestinal absorption [14,15]. In this sense, synergistic combinations between samples of essential oils and honey have recently been investigated [14].

*Eucalyptus globulus* (*E. globulus*) is a medicinal plant belonging to the Myrtaceae family, known for its medicinal properties. Laboratory investigations showed that *E. globulus* essential oil (EGEO) exhibits numerous biological properties, such as antidiabetic, antioxidant, anti-inflammatory, wound healing, antibacterial, etc. [16,17,18,19,20]. Moreover, this species is importantly used by bees to produce honey called *Eucalyptus* honey, which is remarkably used as food but also as a remedy for certain pathologies, including infections and diarrhea. Indeed, *E. globulus* honey is rich in phenolic and flavonoid compounds [21,22,23] and also has demonstrated numerous biological effects, such as antioxidant, antimicrobial, etc. [24,25].

The objectives of this work are the determination of the effects of EGEO as well as the examination of the biological properties, namely, the antioxidant, anti-inflammatory, dermatoprotective, and antimicrobial activities, of EGEO, *E. globulus* honey, and their mixture.

## 2. Materials and Methods

### 2.1. Chemicals and Reagents

2,2′-diphenyl-1-picrylhydrazyl (DPPH), 2,2-azino-bis-3-ethylbenzothiazoline-6-sulfonic acid (ABTS), 6-hydroxy-2,5,7,8-tetramethylchroman-2-carboxylic acid (Trolox), and ascorbic acid were purchased from Sigma-Aldrich (Saint-Quentin-Fallavier, France). Lipoxygenase (5-LOX) and linolenic acid were purchased from Sigma-Aldrich (St. Louis, MO, USA). Mueller–Hinton Agar, DMSO, and chloramphenicol were purchased from (Biokar, Beauvais, France). All other reagents were of analytical grade.

### 2.2. Eucalyptus globulus Essential Oil Extraction and Honey Samples Collection

Honey samples of *E. globulus* and *E. globulus* leaves were provided by beekeepers in Larache province (35°07′21.9″ N 6°09′10.2″ W). The identification of *E. globulus* was carried out at the Scientific Institute, Mohammed V University in Rabat, and a Voucher specimen was given (RAB 113264). The extraction process of *E. globulus* essential oil (EO) was carried out by hydro-distillation in a Clevenger-type apparatus. Briefly, 100 g of the dry plant was placed in a balloon filled to 2/3 with water; the whole was brought to boil for 3 h. The oil was recovered and conserved at a temperature of 4 °C for the tests. The obtained honey samples were stocked at room temperature, and the studies were carried out before exceeding 3 months from the collection date. Multiple methods were applied to estimate the physicochemical properties of the collected honey samples, and all these methods were performed as described by Chakir et al. [26].

### 2.3. Chemical Composition Analysis of Eucalyptus globulus Essential Oil

The chemical components of *E. globulus* EO were determined using gas chromatography coupled to mass spectrometry (GC/MS) analysis conditions as described by Mekkaoui et al. [27]. Briefly, a Hewlett-Packard (HP6890) GC instrument coupled with an HP5973 MS and equipped with a 5% phenylmethyl silicone HP−5 MS capillary column (30 m × 0.25 mm × film thickness 0.25 μm) was used in GC analysis. The column temperature was increased from 50 °C for 5 min to 200 °C with a 4 °C/min rate. Helium with a 1.5 mL/min flow rate and split mode (flow: 112 mL/min, ratio: 1/74.7) was the carrier gas used. The hold time was 48 min, while the temperature of the injector and detector was 250 °C.

The machine was led by the computer system ″HP ChemStation″, managing the functioning of the machine and allowing us to follow the evolution of chromatographic analyses. Diluted samples (1/20 in methanol) of 1 μL were injected manually. In addition, 70 eV ionization voltage, 230 °C ion source temperature, and a 35–450 (*m*/*z*) scanning range were the MS operating conditions. Finally, the qualitative quantification of the different compounds was based on the percent area of each peak of the sample compounds and was confirmed by reference to their MS identities (Library of NIST/EPA/NIH MASS SPECTRAL LIBRARY Version 2.0, build 1 July 2002).

### 2.4. In Vitro Antioxidant Assays

The in vitro antioxidant activities of *E. globulus* EO, *E. globulus* honey, and their combination, were assessed by three complementary methods, namely, DPPH scavenging activity, reducing power, and *β*-carotene/linoleic acid assays.

Ferric Reducing Antioxidant Power Assay

The reducing power of *E. globulus* EO, *E. globulus* honey, and their combination was determined according to the protocol described by Ould Si Said et al. [28] with slight modifications. Phosphate buffer (0.2 M, pH = 6.6) was prepared by mixing Na_2_ HPO_4_ and NaH_2_ PO_4_ in an appropriate ratio. Samples (*E. globulus* EO, *E. globulus* honey, and their combination) in an amount of 1 mL at different dilutions in methanol solvent (10, 20, 30, 40 and 50 mg/mL) were mixed with 1 mL of phosphate buffer and 1 mL of 1% of potassium ferrocyanide K3 Fe(CN)_6_. The obtained mixture was incubated at 50 °C for 20 min. After the incubation, 1 mL of 10% trichloroacetic acid (TCA) was added to the mixture, followed by centrifugation at 3000 rpm for 10 min. Afterwards, the supernatant was mixed with 1.5 mL of distilled water and 150 µL of 0.1% FeCl_3_. The absorbance was measured at 700 nm and compared against BHA, which was used as the reference. A higher value of the absorbance corresponds to an increase in the reducing power. The antioxidant power was expressed as IC_50_ value (mg/mL). Tests were performed in triplicate.

Inhibition of Lipid Peroxidation

The anti-lipid peroxidation capacity was determined by the linoleic acid/β-carotene assay, as described by Ould Si Said et al. [28] with slight modifications. A solution of β-carotene and linoleic acid was prepared as follows: 0.5 mg of β-carotene was dissolved in 1 mL of chloroform, and 25 μL linoleic acid and 200 mg Tween 40 were added. The chloroform was evaporated. Then, 100 mL of distilled water was added. Afterwards, a 2.5 mL aliquot of this reaction mixture was dispensed into test tubes, and 350 μL of the prepared samples at 2 mg/mL was added. The emulsion was incubated for 48 h at room temperature. The same procedure was repeated with butylated hydroxyl anisole (BHA) as a standard and with a blank (without BHA). After 48 h, the absorbance was measured at 490 nm. The antioxidant activity was expressed as IC_50_ value (mg/mL). All samples were tested in triplicate.

Relative antioxidant activity was calculated in the following way:

(1) $$\mathrm{Antioxidant}\;\mathrm{activity}\;\left(\%\right)=\frac{\mathrm{At}}{A0}\times 100$$

where A0 is the absorbance of the test compound (EOs) at the beginning of incubation, and At is the absorbance of the test compound (EOs) at the end of incubation.

DPPH (2,2-Diphenyl−1-picrylhydrazyl) Radical Scavenging Activity

The DPPH test was carried out in order to measure the free radical scavenging activity and following the same procedures as previously described by El Omari [29]. Briefly, various concentrations of the test samples (i.e., *E. globulus* EO, *E. globulus* honey, and their combination (1:1)) were prepared in pure methanol solvent. Then, 1 mL of each sample was added to 0.25 mL of 0.2 mmol/L (*v*/*v*) DPPH radical solution. The reaction mixture was incubated in the dark at room temperature for 30 min. The absorbance was read against a blank (methanol) at 517 nm. BHA (butylated hydroxyl anisole) was used as a positive control. All tests were performed in triplicate. The DPPH radical scavenging activity was calculated according to the following formula:

(2) $$\mathrm{DPPH}\;\left(\%\right)=\frac{\left({\mathrm{Abs}}_{\mathrm{DPPH}}-{\mathrm{Abs}}_{\mathrm{Sample}}\right)}{{\mathrm{Abs}}_{\mathrm{DPPH}}}\times 100\;$$

where Abs_DPPH_ represents the absorbance of the DPPH radical, and Abs_sample_ is the absorbance in the presence of extract/control. The antiradical scavenging activity was expressed as IC_50_ value in mg·mL^−1^.

### 2.5. In Vitro Anti-Inflammatory and Dermatoprotective Assays

The in vitro dermatoprotective effects of *E. globulus* EO, *E. globulus* honey, and their combination were estimated by their capacity to inhibit tyrosinase activity. The test was carried out by following the method described by Bouyahya et al. [30]. On the other hand, the 5-Lipoxygenase (5-LOX) inhibitory activity, based on linoleic acid oxidation at 234 nm, was used to evaluate the in vitro anti-inflammatory effect according to the previously published method [8]. Briefly, 20 µL of *E. globulus* EO, *E. globulus* honey, and their combination (1:1) and 20 µL of 5-LOX from Glycine max (100 U/mL) were pre-incubated with 200 µL of phosphate buffer (0.1 M, pH 9) at room temperature for 5 min. Then, 20 µL of linolenic acid (4.18 mM in ethanol) was added in order to start the reaction, which was followed for 3 min at 234 nm. Each assay was performed in triplicate, and quercetin was used as a positive control.

### 2.6. In Vivo Anti-Inflammatory Assay

The in vivo anti-inflammatory effect was carried out using a rat model of carrageenan-induced paw edema [31]. Briefly, Wistar rats (150 to 180 g) were made to fast for 18 h and then randomly divided into eight groups (*n* = 6 per group): The first six groups were orally administered with two different concentrations (50 and 100 mg/kg) of *E. globulus* EO, *E. globulus* honey, and their combination (1:1), respectively. The seventh group was a negative control that received distilled water, while the last group was considered a positive control and received indomethacin (10 mg/kg) as a reference anti-inflammatory drug. After 60 min, all rats were injected subcutaneously with carrageenan solution (0.05 mL of 1% carrageenan suspended in 0.9% NaCl) into the subplantar region of the left hind paw. The paw volumes of the tested rats were recorded using a LE 7500 digital plethysmometer controlled by SeDaCOM software before the injection of carrageenan (V0) and after the carrageenan injection at three different times: 1 h, 3 h, and 6 h (Vt) [31]. In each group, the anti-inflammatory effect was calculated using the following equation:

% of inhibition= [(*V_t_ − V_0_*)_control_
*−* (*V_t_ − V_0_*)_treated group_]/(*V_t_ − V_0_*)_control_] × 100.

### 2.7. Assessment of Antimicrobial Activity

#### 2.7.1. Tested Microorganisms

The antibacterial activity was evaluated against the following six bacterial strains representing Gram-positive and Gram-negative bacteria: *Escherichia coli* ATCC 25922, *Proteus mirabilis* ATCC 25933, *Salmonella Typhimurium* ATCC 700408, *Bacillus subtilis* ATCC 6633, *Staphylococcus aureus* ATCC 29213, and *Listeria monocytogenes* ATCC 13932. Additionally, the antimicrobial activity was evaluated against one yeast (*Candida albicans*) and two fungi (*Trichophyton rubrum* and *Aspergillus niger*).

#### 2.7.2. Inoculum Preparation

The revivification of bacteria was conducted by cultivation the frozen strains (−20 °C) in Mueller–Hinton agar medium, followed by an incubation at 37 °C for 24 h. Afterward, a bacterial suspension at 0.5 McFarland was prepared in sterile physiologic water. On the other hand, the yeast strain was cultivated in Sabouraud agar medium and incubated at 25 °C. After 48 h of incubation, the yeast suspension at 0.5 McFarland was prepared in sterile physiologic water. Finally, the fungi were sub-cultured in Sabouraud agar medium for 5 days at 25 °C. The obtained culture was washed using sterile physiologic water and transferred to a sterile assay tube, leaving the heavy particle to settle for 5 min. The upper homogeneous suspensions were transferred to a new sterile assay tube and adjusted microscopically to about 10^4^ CFU/mL. The obtained inocula of bacteria, yeast, and fungi were used to evaluate the antimicrobial activity of *E. globulus* essential oils, honey, and their mixture.

#### 2.7.3. Disc Diffusion Assay

The primary screening of the antimicrobial activity of the studied samples was evaluated by the disc diffusion method according to the previously published protocols [32,33]. Briefly, the culture suspension of each species was inoculated in the optimal culture medium (Mueller–Hinton agar for bacteria and Sabouraud agar for yeast and fungi). Afterwards, 6 mm diameter sterile paper discs soaked with 10 µL of Eucalyptus essential oil (mixed with 5% of DMSO), Eucalyptus honey, or the mixture of Eucalyptus essential oil and honey were deposited on each plate. Chloramphenicol (30 µg) was used as positive control for bacteria, and nystatin (100 I.U.) was used as positive control for yeast and fungi, while DMSO (10 µL; 5%) was used as negative control. The plates were incubated at the following growth conditions: 37°C for 24 h, 25 °C for 48 h, and 25 °C for 72 h, for bacteria, yeast, and fungi, respectively. After incubation, the inhibitory diameters were measured in millimeters, and the results were expressed as means ± standard deviation of three replicates.

#### 2.7.4. Determination of Minimum Inhibitory Concentration

The minimum inhibitory concentration (MIC) corresponds to the minimum concentration of a sample that is able to inhibit the growth of microorganisms. In this study, the determination of MIC against bacteria and yeast was performed according to the protocol previously described [32] with some modification. Mueller–Hinton broth (Biokar, Beauvais, France) and Sabouraud broth (Biokar, Beauvais, France) media were used for bacteria and yeast, respectively. The incubation was conducted at 37 °C for 24 h for bacteria and at 25 °C for 48 h for yeast. However, the determination of MIC against the fungi strain was conducted using the gradient plate method according to the protocol previously described [33]. Chloramphenicol was used as positive control for bacteria, while nystatin was used for yeast and fungi.

#### 2.7.5. Determination of Minimum Bactericidal Concentration

Minimum bactericidal concentration (MBC) corresponds to the minimum concentration of a sample that can kill the microorganism. The same microdilution experiment derived from the determination of MIC was used. After the incubation, 10 μL of each tube that did not present visible growth was sub-cultured on Tryptone Soy Agar (Biokar, Beauvais, France) and incubated at 37 °C for 24 h, and the lowest concentration that did not present any growth on media was considered as the MBC [34].

### 2.8. Statistical Analysis

All experiments were conducted in triplicate, and the obtained results are expressed as mean ± SD. Data were analyzed using SPSS software version 21, and comparisons between means were done using one-way ANOVA, followed by Tukey test. Differences between means were considered significant when *p* < 0.05.

## 3. Results

### 3.1. Physicochemical Properties of E. globulus Honey Samples

The physicochemical characteristics of *E. globulus* honey samples tested in this study are summarized in Table 1. As can be seen, the collected samples of honey have an extra light amber color, a moisture rate of 11.63 ± 1.19 %, and a pH value of 4.01 ± 0.02, essentially due to free acidity (23.48 ± 0.04 meq/kg) and lactone acidity (3.41 ± 0.01 meq/kg). The honey samples were also characterized by an electrical conductivity of 0.49 ± 0.03 ms/cm and a density of 1.40 ± 0.02 g/mL.

### 3.2. Chemical Composition of Eucalyptus globulus Essential Oil

**Figure 1:**
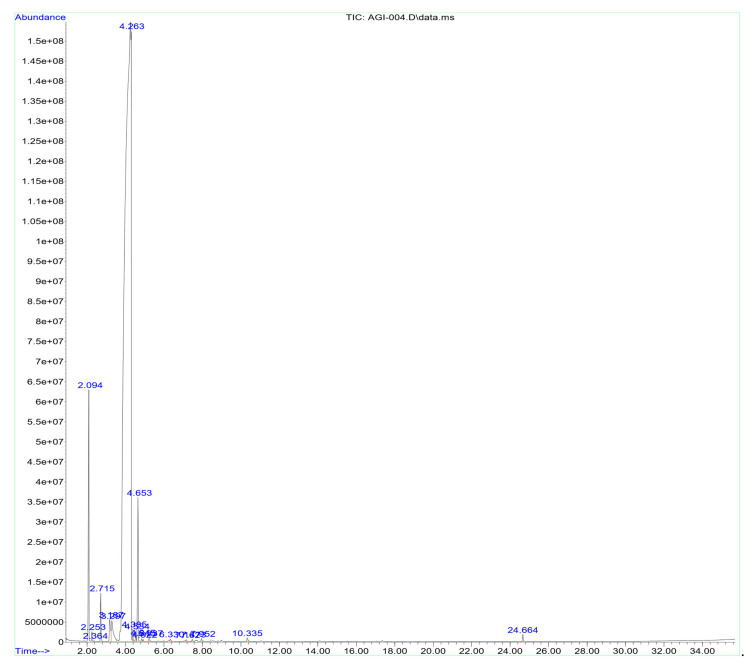
Chromatography analysis of EGEO.

As shown in Figure 1 and Table 2, the major bioactive compound detected in EGEO was eucalyptol (1,8-cineole) (90.14%). Other well-known bioactive compounds, such as *α*-and *β*-pinene (3.85% and 0.62%, respectively), *γ*-terpinene (2.39%), *α*-phellandrene (0.96%), *β*-myrcene (0.58%), and camphene (0.48%), were also detected.

### 3.3. In Vitro Antioxidant Activity

Medicinal plants are a reserve of natural bioactive substances possessing several antioxidant properties in different biological systems. In the present study, the antioxidant effects of EGEO, *E. globulus* honey, and their mixture were, evaluated using three methods, namely DPPH, reducing power, and *β*-carotene/linoleic acid assays. The results are shown in Table 3, and the IC_50_ values were calculated to compare these results to those of BHA, which was used as a reference antioxidant. According to the recorded results, all samples had the capacity to reduce the stable violet DPPH radical to yellow DPPH-H, with the 50% reduction values IC_50_ = 0.37 ± 0.06 mg/mL, IC_50_ = 0.28 ± 0.12 mg/mL, and IC_50_ =0.19 ± 0.08 mg/mL for EGEO, *E. globulus* honey, and their mixture, respectively. However, when compared to the pure reference antioxidant BHA (0.5 ± 0.01 mg/mL), all the tested samples showed a lower antioxidant activity. Furthermore, all the assessed samples were able to reduce the ferulic ions in the reducing power assay, with IC_50_ = 0.29 ± 0.03 mg/mL, IC_50_ = 0.17 ± 0.06 mg/mL, and 0.08 ± 0.01 mg/mL for EGEO, *E. globulus* honey, and their mixture, respectively. However, these values were less important when compared to the pure reference antioxidant BHA (0.03 ± 0.05 mg/mL).

The inhibition of the lipid peroxidation activity was assayed by the *β*-carotene-bleaching test. The activity of the samples was found to be dose-dependent, with IC_50_ values of 0.17 ± 0.01 mg/mL, 0.11 ± 0.02 mg/mL, and 0.07 ± 0.05 mg/mL for the tested EGEO, *E. globulus* honey, and their mixture, respectively. These values were also higher than that of the standard BHA (0.05 ± 0.03 mg/mL). Compared to the DPPH scavenging effect and the reducing power, EGEO was more active in the inhibition of the lipid peroxidation, presumably due to the high specificity of the test for lipophilic compounds.

### 3.4. In Vitro Anti-Inflammatory and Dermatoprotective Activities

Inflammation is a natural reaction of vascular tissues to a causative agent responsible for the activation of phospholipase A_2_ and, subsequently, the production of arachidonic acid and inflammatory mediators, accelerating the movement of leukocytes to the inflammation site [35]. In this study, we investigated the anti-inflammatory and dermatoprotective activity of EGEO and *E. globulus* honey as well as their combination using the 5-LOX and tyrosinase enzyme inhibition assay. The obtained results are represented in Table 4.

Concerning the in vitro test, the highest activity was recorded with EGEO (IC_50_ = 0.88 ± 0.01 μg/mL) compared to honey (IC_50_ = 4.23 ± 0.07 μg/mL), while their combination showed a synergistic effect (IC_50_ = 1.02 ± 0.03 μg/mL). These values were highly remarkable compared to the reference compound, namely, quercetin (IC_50_ = 0.09 ± 0.05 μg/mL).

The dermatoprotective activity of EGEO and *E. globulus* honey as well as their mixture was investigated by testing their inhibitory effect on tyrosinase (an enzyme activating the oxidation of tyrosine, leading to melanin secretion).

The results showed that EGEO exhibited the best activity (IC_50_ = 38.21 ± 0.13 μg/mL) compared to honey (IC_50_ = 79.13 ± 0.05 μg/mL), while the combination between them provided a significant synergistic effect with an IC_50_ of 41.32 ± 0.01 μg/mL. These results were promising compared to quercetin (IC_50_ = 45.68 ± 0.02 μg/mL) used as a standard molecule.

### 3.5. In Vivo Anti-Inflammatory Activity

The anti-inflammation effect is a crucial physiological defensive mechanism against several complicated diseases, such as diabetes, cancer, and autoimmune and neurodegenerative disorders [36]. It has been shown that the use of synthetic anti-inflammatory drugs causes several side effects on the human body. This problem has increased the interest in searching for new alternatives, especially from natural products. The latter are considered an important source of bioactive molecules with anti-inflammatory activity [8]. Natural products, such as honey and essential oils, are one of the best targets for these molecules since they do not produce any side effects. Indeed, the effects of *E. globulus* essential oil, honey, and their mixture at 50 and 100 mg/kg (test doses) on carrageenan-induced acute inflammation, estimated by the increased paw volume of the rats at three different time periods (1, 3, and 6 h), are presented in Table 5. The results showed that the injection of carrageenan into the subplantar tissue of the control groups of rats induced edema development, which peaked at 1.63 mL in paw volume after 6 h of the experimental induced paw edema. The latter confirmed that the experimental carrageenan injection provoked a local and acute inflammatory reaction [37]. In this model, EGEO at 50 and 100 mg/kg p.o. exhibited maximum anti-inflammatory activity, with 59%, 25%, and 69.13% edema inhibition after 1 h, 3 h, and 6 h, respectively. Indeed, after 6 h, EGEOnat 100 mg/kg showed an inhibition of 69.13%, which was more important than that observed with indomethacin at 10 mg/kg, p.o. (64.19%). Moreover, the effect of honey on carrageenan-induced rat paw edema was dose-dependent from the first to sixth hours after carrageenan injection, with a peak effect of 61.72% produced at 100 mg/kg after 6 h. The combination of EGEO and honey showed considerable anti-inflammatory activity, with an inhibition of 30.76%, 59.42%, and 71.60% produced at 1 h, 3 h, and 6 h, respectively. The results were comparable with the reduction produced by 10 mg/kg of indomethacin, a standard drug, at 1 h, 3 h, and 6 h, which presented inflammation inhibition values of 48.07%, 57.97%, and 64.19%, respectively.

### 3.6. Antimicrobial Activity

The antimicrobial activity of EGEO and *E. globulus* honey has been considered to be one of their most relevant biological properties [38]. In this study, the antimicrobial effect of EGEO, *E. globulus* honey, and their mixture was determined against six bacterial species, one yeast, and two fungal strains by the disc diffusion and microdilution methods. The results compared to those of chloramphenicol and nystatin (used as positive controls) are summarized in Table 6 and Table 7.

The antibacterial activity of *E. globulus* honey using the disc diffusion method showed a remarkable activity against the tested bacteria, with inhibition zones ranging from 8.0 ± 0.0 mm to 11.1 ± 0.2, and against yeast and fungal strains with an inhibitory diameter of 6.0 ± 0.0 mm. The largest bacterial inhibitory zone for *E. globulus* honey was observed against Gram-positive bacteria. Thus, *L. monocytogenes* was the most sensitive bacterial strain, while *S. typhimurium* was the most resistant compared to other species. Concerning the standard agents, chloramphenicol showed inhibitory zones ranging from 16.0 ± 0.1 mm to 28.9 ± 0.2 mm and nystatin from 25.7 ± 0.1 mm and 29.0 ± 0.1 mm. El-Borai et al. [39] evaluated the antibacterial activity of *E. globulus* honey and showed larger inhibitory zones compared to our study [39]. In fact, the reported inhibitory zones were 13.67 mm and 35.67 mm against *E. coli* and *P. mirabilis,* respectively.

The minimum inhibitory concentrations and the minimum bactericidal concentrations are cited by most researchers as a measure of the antibacterial performance of a product. In this study, the MIC and MBC values fluctuated between 4 and 8%.

The tested essential oil of *Eucalyptus* in the agar plates against the studied Gram-negative and Gram-positive bacterial strains showed inhibitory diameters that ranged from 14.1 ± 0.1 mm to 21.4 ± 0.1 mm. The smallest inhibitory zone was obtained against *S. typhimurium* (14.1 ± 0.1 mm), while the largest one was observed against *L. monocytogenes* (21.4 ± 0.1 mm). Generally, larger inhibitory zone diameters correlated with lower MIC values. EGEO showed a strong antimicrobial effect against the majority of the selected microorganisms. MIC values ranged between 0.5% and 2%, with the highest activity against *B. subtilis*, *S. aureus*, and *L. monocytogenes,* while the lowest effect was observed against *P. mirabilis* and *E. coli.* EGEO also exhibited strong activity against yeast and fungal strains, with inhibitory zones that ranged from 12.9 ± 0.1 mm to 16.9 ± 3.2 mm and MIC values that varied from 2% to 4%. These results confirmed the potential application of *Eucalyptus globulus* essential oil in the food matrices and pharmaceutical industries, which may be used as an alternative antifungal and antibacterial agent for the treatment of several infectious diseases [40]. Furthermore, the combination of honey and essential oil reduced the inhibitory zones and MICs of the essential oil for all strains. The weakest synergism was obtained with the mixture against *S. typhimurium*, with an inhibitory zone of 10.4 ± 0.1 mm and MIC value of 8%. However, *L. monocytogenes* showed a much greater inhibitory zone (15.1 ± 0.1 mm) and a lower MIC value (2%).

## 4. Discussion

Here, we reported the biological effects of EGEO and *E. globulus* honey, as well as the association between these two natural products. Following in vitro and in vivo approaches, remarkable results were obtained and significantly revealed the importance of honey and EO of *E. globulus* as natural sources of biologically bioactive compounds.

Firstly, the essential physicochemical characteristics of *E. globulus* honey, such as color, moisture rate, pH, electrical conductivity, and density, were determined. The results of these analyses showed some differences with other samples of *E. globulus* honey provided from other regions [22,23]. Certainly, the physicochemical characteristics of honey depend mainly on the total composition of honey, which also depends on the geographical origin of the samples. Indeed, the literature has shown an important variability of honey composition and, therefore, the physicochemical characteristics according to different regions [41].

The phytochemical investigations of EGEO using GC-MS analysis showed the presence of 1,8-cineole as the main component. These findings corroborate importantly with those obtained by several research studies investigating the chemical composition of *E. globulus*, with great similarity in the majority of the compounds identified [42,43,44,45,46,47]. However, some differences should be noted between the chemical compounds revealed in this study and those identified in other regions [48,49]. The difference can be explained by several factors that influence the synthesis and the secretion of EO compounds in aromatic plants.

Using three complementary methods, the EO and honey of *E. globulus* showed important antioxidant effects. Compared to previous studies, the IC_50_ values of the honey sample tested in the present study were found to be lower than that of Egyptian honey (37.62 mg/mL, 2.67 mg/mL, and 19.46 mg/mL for DPPH, RP (reducing power), and β-carotene tests, respectively) [39], Brazilian honey (25.4 mg/mL for DPPH) [50], and honey from Turkey (24.43 ± 1.54 mg/mL for DPPH) [51], suggesting the high quality of Moroccan Eucalyptus honey, as represented by their high antioxidant potentials. Furthermore, the essential oil in the present study showed stronger antioxidant activity compared to the results reported for the essential oil of Algerian *E. globulus* leaves with an IC_50_ of 33.33 ± 0.55 mg/mL, 115.39 ± 1.45 mg/mL, and 6.753 ± 0.39 mg/mL for DPPH, RP, and LP, respectively [45]. Previous studies indicated that the antioxidant potential of honey is due to the presence of bioactive compounds, such as polyphenols and flavonoids, and varies widely depending on floral source and geographical origin [52,53]. Moreover, it has been previously reported that 1,8-cineole exhibits various degrees of reducing power, radical scavenging, and chelating in addition to its DNA-protection capacity [46]. Thus, the strong antioxidant activity of eucalyptus essential oils could be mainly due to the presence of 1,8-cineole in a high quantity (90.14%). In addition, the antioxidant activity of EOs was attributed also to their terpenes content, such as α-pinene, β-pinene, β-myrcene, and γ-terpinene, which have been known to have good antioxidant properties [16]. According to these results, the combination between the honey and essential oil of *Eucalyptus* presented the best antioxidant capacity and also had the capacity to inhibit lipid peroxidation, which may be due to the synergistic effects of their compounds. These results give this mixture an important potential in combating oxidative damage and in preventing the pathogenesis of many diseases caused by reactive oxygen species, such as cancer, coronary disease, and neurological degeneration, as well as in food conservation, since the unwanted side effects of synthetic antioxidants are widely known, namely, liver damage and carcinogenesis [54].

The anti-inflammatory activity was determined using in vitro and in vivo tests. The in vitro effect revealed an important inhibition of 5-LOX. Using the same assay, other works have already investigated the 5-LOX inhibitory activity of *E. globulus* EO and showed IC_50_ values of 0.16 ± 0.07 mg/mL and 58 ± 1.4 μg/mL [55] [56]. Moreover, a methanolic extract of *E. globulus* showed an inhibition of 78.9% [57]. This has also been confirmed by other in vitro methods. Indeed, Vigo et al. [58] evaluated the effect of this plant on nitric oxide (NO) production in the murine macrophage J774 A.1 cell line [58]. In fact, NO is implicated in several inflammatory pathologies. In addition, a dose-dependent inhibition of protein denaturation was obtained with *E. globulus* EOs and extracts using egg and bovine serum albumin methods [59,60].

Moreover, both the EO and honey of *E. globulus* showed remarkable anti-inflammatory activities. These effects could be attributed to the presence of chemical constituents, such as flavonoids, in honey, which has been widely reported to inhibit the cyclooxygenase and lipoxygenase pathways of arachidonate metabolism [61]. They have also been shown to inhibit the release of proinflammatory cytokines TNF-α and IL−1β, as well as to downregulate the expression of inducible nitric oxide synthase (iNOS) and the production of ROS. However, other studies have previously shown that the monoterpene components of *E. globulus* EO, such as 1,8-cineole, are potent inhibitors of inflammatory mediators (cytokines) and down-regulators of the production of leukotriene B2, prostaglandin E2, and other arachidonic acid metabolites in human monocytes [62]. In addition, several investigators reported that *α*-pinene exhibits anti-inflammatory activity through numerous mechanisms, such as a decrease in expression levels of proinflammatory cytokines, TNF-α and IL−1β, and cyclooxygenase [63,64].

On the other hand, dermato-protection was investigated by the inhibitory effect of tyrosinase and showed interesting results. The outcomes of this finding were in agreement with those obtained by Sugimoto et al. [65], who noted a remarkable inhibitory activity of *E. globulus* leaves hydro-alcoholic extract (IC_50_ = 0.39 mg/mL). In a very recent study, Moreira et al. [66] investigated the protective effect of the essential oil and hydro-distillation residual water extract of *E. globulus* leaves against skin damage. Indeed, they observed a reduction in age-related markers of senescence, such as the upregulation of type I collagen and the activation of matrix metalloproteinases and β-galactosidase. Inhibition of melanin and tyrosinase production has also been noted. In parallel, all these findings have been confirmed by in vivo studies following various experimental protocols. In 2011, Bhatt and colleagues tested the effectiveness of eucalyptus EO in controlling acne via the inhibition of sebum synthesis from the sebaceous glands [67]. Using a model of rat sebaceous glands, a decrease in the size of these latter was noticed, and consequently, acne spreading was inhibited.

On the other hand, several researchers have evaluated the dermatoprotective potential of *E. globulus* by evaluating its wound repair capacity [19,68]. Indeed, Tomen and colleagues showed a significant wound-healing effect with *E. globulus* fruit EO in rats using incision and excision wound models [19]. In another recent study, a second-degree wound was induced in rats and coated with tissue prepared from *E. globulus* leaf extracts. Wound closure measurement showed that the extracts of this plant improve wound healing [68].

Infectious diseases caused by different microorganisms such as bacteria and fungi mainly contribute as risk factors in the genesis of some complex pathologies, such as cancer, chronic inflammation, and diabetes. Moreover, the identification of bioactive compounds with antimicrobial effects is considered a promising way to reduce and prevent complex and systematic pathologies. In this way, the EO and honey of *E. globulus* were tested for their antimicrobial properties against some bacterial and fungal strains. As findings, significant results were revealed at low concentrations, and a synergistic action was also found between the EO and honey of *E. globulus.* Several reports have assessed and confirmed the in vitro antibacterial, anti-*candida*, and antifungal potential of the essential oil and honey of *E. globulus* [23,40,46,69,70,71]. These studies reported that the antibacterial activity of honey is related to the polyphenol compounds, hydrogen peroxide content, peptide Bee defensin−1, and methylglyoxal (MGO), as well as its osmotic properties, its acidity, HMF, low pH, and other physicochemical parameters [23]. Moreover, the authors demonstrated that this activity varies according to botanical and geographical origin as well as climatic conditions [39]. On the other hand, a number of researchers have shown that the antimicrobial activity of *Eucalyptus* EO are attributed to the oxygenated monoterpenes, such as 1,8-cineole, α-pinene, and β-pinene, which are well-known constituents with pronounced antimicrobial activities [72]. These compounds have been shown to disintegrate the outer membrane of bacteria, releasing lipopolysaccharides and increasing the permeability of the cytoplasmic membrane to ATP [71].

## 5. Conclusions

The investigation of some biological activities of EGEO, honey, and their mixture confirmed the pharmacological potential of this plant. The strong antioxidant, anti-inflammatory, and dermatoprotective effects make the latter a promising target for alternative drugs against degenerative diseases. In addition, the bactericidal, fungicidal, and candidacidal activities of *E. globulus* shown in this work confirm the antimicrobial potential of this plant.

## Figures and Tables

**Table 1:** Physicochemical characteristics of *E. globulus* honey.

Parameter	Description
Color	Extra Light Amber
Moisture (%)	11.63 ± 1.19
pH	4.01 ± 0.02
Free acidity (meq/kg)	23.48 ± 0.04
HMF (mg/kg)	11.24 ± 0.05
Lactone acidity (meq/kg)	3.41 ± 0.01
Electrical conductivity (ms/cm)	0.49 ± 0.03
Density (g/mL)	1.40 ± 0.02
Ashes (%)	0.37 ± 0.01

**Table 2:** Chemical composition of EGEO.

No	Compound	%
1	**eucalyptol (1,8-cineole)**	90.14
2	***α*-pinene**	3.85
3	***γ*-terpinene**	2.39
6	***α*-phellandrene**	0.96
7	***β*-pinene**	0.62
8	***β*-myrcene**	0.58
9	**Camphene**	0.48
10	***β*-ocimene**	0.28

**Table 3:** The antioxidant activity of EGEO, *E. globulus* honey, and their mixture (IC_50_ in μg/mL).

Samples	DPPH	RP	LP
**Essential oil**	0.37 ± 0.06 ^ns^	0.29 ± 0.03 ***	0.17 ± 0.01 **
**Honey**	0.28 ± 0.12 *	0.17 ± 0.06 *	0.11 ± 0.02 ^ns^
**Mixture**	0.19 ± 0.08 **	0.08 ± 0.01 ^ns^	0.07 ± 0.05 ^ns^
**BHA**	0.5 ± 0.01	0.05 ± 0.03	0.05 ± 0.03

Values are mean ± SEM (*n* = 3). * *p* < 0.05, ** *p* < 0.01, *** *p* < 0.001, and ns = not significant compared to BHA. DPPH: 2,2-diphényl 1-picrylhydrazyle; RP: Reducing Power; LP: Inhibition of Lipid Peroxidation.

**Table 4:** In vitro anti-inflammatory and dermatoprotective activity.

Assay	EGEO (IC_50_ μg/mL)	*E. globulus* Honey (IC_50_ μg/mL)	EGEO/Honey Mixture (IC_50_ μg/mL)	Quercetin (IC_50_ μg/mL)
**5-Lipoxygenase**	0.88 ± 0.01 ****	4.23 ± 0.07 ****	1.02 ± 0.03 ****	0.09 ± 0.05
**Tyrosinase**	38.21 ± 0.13 ****	79.13 ± 0.05 ****	41.32 ± 0.01 ****	45.68 ± 0.02

Values are mean ± SEM (*n* = 3). **** *p* < 0.0001.

**Table 5:** Inhibition percentage of the left hind paw volume in rats treated with EGEO, *E. globulus* honey, and their mixture.

Drugs	Dose (mg/kg)	Carrageenan-Induced Hind Paw Edema Volume (mL mean s.e.m.) and % of Inhibition						
		**T0**	**1 h**	**% inh**	**3 h**	%inh	6 h	% inh
Control	-	0.82	1.34		1.51		1.63	
EGEO	50	0.79	1.22	17.30	1.18	43.47	1.12	59.25
	100	0.73	1.13	23.07	1.04	55.07	0.98	69.13
*E. globulus* honey	50	0.82	1.26	15.38	1.22	42.02	1.20	53.08
	100	0.83	1.25	19.23	1.20	46.37	1.14	61.72
EGEO and *E. globulus* honey mixture (1/1)	50	0.80	1.18	26.92	1.15	49.27	1.12	60.49
	100	0.84	1.20	30.76	1.12	59.42	1.07	71.60
Indomethacin	10	0.84	1.11	48.07	1.13	57.97	1.13	64.19

**Table 6:** Mean zones of inhibition of *E. globulus* EO, *E. globulus* honey, and mixture (mean of three replicates ± standard deviations).

Microorganisms	Samples			Controls	
	EGEO	*E. globulus* Honey	Mixture	Chloramphenicol	Nystatin
*E. coli* ATCC 25922	17.4 ± 0.1 ****	10.0 ± 0.1 ****	13.3 ± 0.1 ****	22.4 ± 0.0	nt
*P. mirabilis* ATCC 25933	16.9 ± 0.1 ****	9.8 ± 0.1 ****	13.0 ± 0.1 ****	22.4 ± 0.1	nt
*S. typhimurium* ATCC 700408	14.1 ± 0.1 ****	8.0 ± 0.0 ****	10.4 ± 0.1 ****	13.2 ± 0.1	nt
*B. subtilis* ATCC 6633	21.1 ± 0.1 ****	11.1 ± 0.2 ****	14.5 ± 0.2 ****	16.0 ± 0.1	nt
*S. aureus* ATCC 29213	20.4 ± 0.2 ****	10.6 ± 0.1 ****	14.9 ± 0.1 ****	25.2 ± 0.1	nt
*L. monocytogenes* ATCC 13932	21.4 ± 0.1 ****	11.0 ± 0.1 ****	15.1 ± 0.1 ****	28.9 ± 0.2	nt
*Candida albicans*	14.8 ± 0.1 ****	6.0 ± 0.0 ****	9.8 ± 0.1 ****	NT	29.0 ± 0.1
*Trichophyton rubrum*	12.9 ± 0.1 ****	6.0 ± 0.0 ****	9.4 ± 0.1 ****	NT	25.7 ± 0.1
*Aspergillus niger*	16.9 ± 3.2 ***	6.0 ± 0.0 ****	8.8 ± 0.2 ****	NT	26.4 ± 0.1

Values are mean ± SEM (*n* = 3). *** *p* < 0.001, **** *p* < 0.0001; NT: not tested.

**Table 7:** MIC and MBC of EGEO, *E. globulus* honey, and their mixture in percentage (*v*/*v*).

Microorganisms	Samples % (*v/v*)						Controls (µg/mL)	
	EGEO		*E. globulus* Honey		Mixture		Chloramphenicol	Nystatin
	MIC	MBC	MIC	MBC	MIC	MBC	MIC	MIC
*E. coli* ATCC 25922	1	1	8	>8	2	4	4	NT
*P. mirabilis* ATCC 25933	1	1	8	>8	2	4	4	NT
*S*. *typhimurium* ATCC 700408	2	2	>8	>8	8	8	64	NT
*B. subtilis* ATCC 6633	0.5	0.5	4	8	1	2	32	NT
*S. aureus* ATCC 29213	0.5	0.5	4	8	1	2	4	NT
*L. monocytogenes* ATCC 13932	0.5	0.5	4	8	1	2	2	NT
*Candida albicans*	2	NT	>8	NT	8	NT	NT	4
*Trichophyton rubrum*	4	NT	>8	NT	8	NT	NT	16
*Aspergillus niger*	4	NT	>8	NT	8	NT	NT	16

NT: not tested.

## Data Availability

Not applicable.

## References

[1] El Baaboua A., El Maadoudi M., Bouyahya A., Belmehdi O., Kounnoun A., Zahli R., Abrini J. (2018). Evaluation of Antimicrobial Activity of Four Organic Acids Used in Chicks Feed to Control Salmonella Typhimurium: Suggestion of Amendment in the Search Standard. Int. J. Microbiol..

[2] Abdelaali B., El Menyiy N., El Omari N., Benali T., Guaouguaou F.-E., Salhi N., Naceiri Mrabti H., Bouyahya A. (2021). Phytochemistry, Toxicology, and Pharmacological Properties of Origanum Elongatum. Evid. Based Complement. Alternat. Med..

[3] Bouyahya A., Chamkhi I., Benali T., Guaouguaou F.-E., Balahbib A., El Omari N., Taha D., Belmehdi O., Ghokhan Z., El Menyiy N. (2021). Traditional Use, Phytochemistry, Toxicology, and Pharmacology of Origanum Majorana L.. J. Ethnopharmacol..

[4] Balahbib A., El Omari N., Hachlafi N.E., Lakhdar F., El Menyiy N., Salhi N., Mrabti H.N., Bakrim S., Zengin G., Bouyahya A. (2021). Health Beneficial and Pharmacological Properties of P-Cymene. Food Chem. Toxicol..

[5] Bouyahya A., Mechchate H., Benali T., Ghchime R., Charfi S., Balahbib A., Burkov P., Shariati M.A., Lorenzo J.M., Omari N.E. (2021). Health Benefits and Pharmacological Properties of Carvone. Biomolecules.

[6] Bouyahya A., Bakri Y., Et-Touys A., Assemian I.C.C., Abrini J., Dakka N. (2018). In Vitro Antiproliferative Activity of Selected Medicinal Plants from the North-West of Morocco on Several Cancer Cell Lines. Eur. J. Integr. Med..

[7] Bouyahya A., El Omari N., Elmenyiy N., Guaouguaou F.-E., Balahbib A., Belmehdi O., Salhi N., Imtara H., Mrabti H.N., El-Shazly M. (2021). Moroccan Antidiabetic Medicinal Plants: Ethnobotanical Studies, Phytochemical Bioactive Compounds, Preclinical Investigations, Toxicological Validations and Clinical Evidences; Challenges, Guidance and Perspectives for Future Management of Diabetes Worldwide. Trends Food Sci. Technol..

[8] Bouyahya A., Guaouguaou F.-E., El Omari N., El Menyiy N., Balahbib A., El-Shazly M., Bakri Y. (2022). Anti-Inflammatory and Analgesic Properties of Moroccan Medicinal Plants: Phytochemistry, In Vitro and In Vivo Investigations, Mechanism Insights, Clinical Evidences and Perspectives. J. Pharm. Anal..

[9] Sharifi-Rad J., Dey A., Koirala N., Shaheen S., El Omari N., Salehi B., Goloshvili T., Silva N.C.C., Bouyahya A., Vitalini S. (2021). Cinnamomum Species: Bridging Phytochemistry Knowledge, Pharmacological Properties and Toxicological Safety for Health Benefits. Front. Pharmacol..

[10] Bouyahya A., Abrini J., Bakri Y., Dakka N. (2016). Essential Oils as Anticancer Agents: News on Mode of Action. Phytothérapie.

[11] Bouyahya A., Belmehdi O., Abrini J., Dakka N., Bakri Y. (2019). Chemical Composition of Mentha Suaveolens and Pinus Halepensis Essential Oils and Their Antibacterial and Antioxidant Activities. Asian Pac. J. Trop. Med..

[12] Oryan A., Alemzadeh E., Moshiri A. (2016). Biological Properties and Therapeutic Activities of Honey in Wound Healing: A Narrative Review and Meta-Analysis. J. Tissue Viability.

[13] Suntiparapop K., Prapaipong P., Chantawannakul P. (2012). Chemical and Biological Properties of Honey from Thai Stingless Bee (Tetragonula Leaviceps). J. Apic. Res..

[14] Bouyahya A., Abrini J., Et-Touys A., Lagrouh F., Dakka N., Bakri Y. (2018). Analyse Phytochimique et Évaluation de l’activité Antioxydante Des Échantillons Du Miel Marocain. Phytothérapie.

[15] Kuzyšinová K., Mudroňová D., Toporčák J., Molnár L., Javorskỳ P. (2016). The Use of Probiotics, Essential Oils and Fatty Acids in the Control of American Foulbrood and Other Bee Diseases. J. Apic. Res..

[16] Belkhodja M., Meddah B., Sidelarbi K., Bouhadi D., Medjadel B., Brakna A. (2022). In Vitro and In Vivo Anti-Inflammatory Potential of Eucalyptus Globulus Essential Oil. Eur. J. Biol. Res..

[17] Ridaoui K., Guenaou I., Taouam I., Cherki M., Bourhim N., Elamrani A., Kabine M. (2022). Comparative Study of the Antioxidant Activity of the Essential Oils of Five Plants against the H2 O2 Induced Stress in Saccharomyces Cerevisiae. Saudi J. Biol. Sci..

[18] Sharma A.D., Kaur I., Singh N. (2021). Synthesis, Characterization, and In Vitro Drug Release and In Vitro Antibacterial Activity of O/W Nanoemulsions Loaded with Natural Eucalyptus Globulus Essential Oil. Int. J. Nanosci. Nanotechnol..

[19] Tomen I., Guragac F.T., Keles H., Reunanen M., Kupeli-Akkol E. Characterization and Wound Repair Potential of Essential Oil Eucalyptus Globulus Labill. Proceedings of the 9 th Annual European Pharma Congress.

[20] Usman L.A., Oguntoye O.S., Ismaeel R.O. (2020). Effect of Seasonal Variation on Chemical Composition, Antidiabetic and Antioxidant Potentials of Leaf Essential Oil of Eucalyptus Globulus L.. J. Essent. Oil Bear. Plants.

[21] Alissandrakis E., Tarantilis P.A., Pappas C., Harizanis P.C., Polissiou M. (2011). Investigation of Organic Extractives from Unifloral Chestnut (Castanea Sativa L.) and Eucalyptus (Eucalyptus Globulus Labill.) Honeys and Flowers to Identification of Botanical Marker Compounds. LWT-Food Sci. Technol..

[22] Bobis O., Moise A.R., Ballesteros I., Reyes E.S., Durán S.S., Sánchez-Sánchez J., Cruz-Quintana S., Giampieri F., Battino M., Alvarez-Suarez J.M. (2020). Eucalyptus Honey: Quality Parameters, Chemical Composition and Health-Promoting Properties. Food Chem..

[23] Valdés-Silverio L.A., Iturralde G., García-Tenesaca M., Paredes-Moreta J., Narváez-Narváez D.A., Rojas-Carrillo M., Tejera E., Beltrán-Ayala P., Giampieri F., Alvarez-Suarez J.M. (2018). Physicochemical Parameters, Chemical Composition, Antioxidant Capacity, Microbial Contamination and Antimicrobial Activity of *Eucalyptus* Honey from the Andean Region of Ecuador. J. Apic. Res..

[24] Malika N., Mohamed F., Chakib E.A. (2005). Microbiological and Physicochemical Properties of Moroccan Honey. Int. J. Agric. Biol..

[25] Zayadi R.A., Bakar F.A., Ahmad M.K. (2019). Elucidation of Synergistic Effect of Eucalyptus Globulus Honey and Zingiber Officinale in the Synthesis of Colloidal Biogenic Gold Nanoparticles with Antioxidant and Catalytic Properties. Sustain. Chem. Pharm..

[26] Chakir A., Romane A., Marcazzan G.L., Ferrazzi P. (2016). Physicochemical Properties of Some Honeys Produced from Different Plants in Morocco. Arab. J. Chem..

[27] Mekkaoui M., Assaggaf H., Qasem A., El-Shemi A., Abdallah E.M., Bouidida E.H., Naceiri Mrabti H., Cherrah Y., Alaoui K. (2021). Ethnopharmacological Survey and Comparative Study of the Healing Activity of Moroccan Thyme Honey and Its Mixture with Selected Essential Oils on Two Types of Wounds on Albino Rabbits. Foods.

[28] Said Z.B.-O.S., Haddadi-Guemghar H., Boulekbache-Makhlouf L., Rigou P., Remini H., Adjaoud A., Khoudja N.K., Madani K. (2016). Essential Oils Composition, Antibacterial and Antioxidant Activities of Hydrodistillated Extract of Eucalyptus Globulus Fruits. Ind. Crops Prod..

[29] El Omari K., Hamze M., Alwan S., Osman M., Jama C., Chihib N.-E. (2019). In-Vitro Evaluation of the Antibacterial Activity of the Essential Oils of Micromeria Barbata, Eucalyptus Globulus and Juniperus Excelsa against Strains of Mycobacterium Tuberculosis (Including MDR), Mycobacterium Kansasii and Mycobacterium Gordonae. J. Infect. Public Health.

[30] Bouyahya A., Belmehdi O., El Jemli M., Marmouzi I., Bourais I., Abrini J., Faouzi M.E.A., Dakka N., Bakri Y. (2019). Chemical Variability of Centaurium Erythraea Essential Oils at Three Developmental Stages and Investigation of Their In Vitro Antioxidant, Antidiabetic, Dermatoprotective and Antibacterial Activities. Ind. Crops Prod..

[31] Rege M.G., Ayanwuyi L.O., Zezi A.U., Odoma S. (2021). Anti-Nociceptive, Anti-Inflammatory and Possible Mechanism of Anti-Nociceptive Action of Methanol Leaf Extract of Nymphaea Lotus Linn (*Nymphaeceae*). J. Tradit. Complement. Med..

[32] Ed-Dra A., Filali F.R., Lo Presti V., Zekkori B., Nalbone L., Bouymajane A., Trabelsi N., Lamberta F., Bentayeb A., Giuffrida A. (2020). Chemical Composition, Antioxidant Capacity and Antibacterial Action of Five Moroccan Essential Oils against Listeria Monocytogenes and Different Serotypes of Salmonella Enterica. Microb. Pathog..

[33] Hu F., Tu X.F., Thakur K., Hu F., Li X.L., Zhang Y.S., Zhang J.G., Wei Z.J. (2019). Comparison of Antifungal Activity of Essential Oils from Different Plants against Three Fungi. Food Chem. Toxicol..

[34] Ed-Dra A., Nalbone L., Filali F.R., Trabelsi N., El Majdoub Y.O., Bouchrif B., Giarratana F., Giuffrida A. (2021). Comprehensive Evaluation on the Use of Thymus Vulgaris Essential Oil as Natural Additive against Different Serotypes of Salmonella Enterica. Sustain. Switz..

[35] Falcão H.d.S., Lima I.O., dos Santos V.L., Dantas H.d.F., Diniz M.d.F., Barbosa-Filho J.M., Batista L.M. (2005). Review of the Plants with Anti-Inflammatory Activity Studied in Brazil. Rev. Bras. Farmacogn..

[36] El Hachimi F., Alfaiz C., Bendriss A., Cherrah Y., Alaoui K. (2017). Activite Anti-Inflammatoire de l’huile Des Graines de *Zizyphus Lotus* (L.) Desf. Phytotherapie.

[37] Boughton-Smith N.K., Deakin A.M., Follenfant R.L., Whittle B.J., Garland L.G. (1993). Role of Oxygen Radicals and Arachidonic Acid Metabolites in the Reverse Passive Arthus Reaction and Carrageenin Paw Oedema in the Rat. Br. J. Pharmacol..

[38] Imtara H., Elamine Y., Lyoussi B. (2018). Honey Antibacterial Effect Boosting Using *Origanum Vulgare* L. Essential Oil. Evid. Based Complement. Alternat. Med..

[39] ElBorai A. (2018). Antibacterial and Antioxidant Activities of Different Varieties of Locally Produced Egyptian Honey. Egypt. J. Bot..

[40] Boukhatem M.N., Boumaiza A., Nada H.G., Rajabi M., Mousa S.A. (2020). Eucalyptus Globulus Essential Oil as a Natural Food Preservative: Antioxidant, Antibacterial and Antifungal Properties In Vitro and in a Real Food Matrix (Orangina Fruit Juice). Appl. Sci..

[41] Faustino C., Pinheiro L. (2021). Analytical Rheology of Honey: A State-of-the-Art Review. Foods.

[42] Adenubi O.T., Abolaji A.O., Salihu T., Akande F.A., Lawal H. (2021). Chemical Composition and Acaricidal Activity of Eucalyptus Globulus Essential Oil against the Vector of Tropical Bovine Piroplasmosis, Rhipicephalus (*Boophilus*) Annulatus. Exp. Appl. Acarol..

[43] Ait-Ouazzou A., Lorán S., Bakkali M., Laglaoui A., Rota C., Herrera A., Pagán R., Conchello P. (2011). Chemical Composition and Antimicrobial Activity of Essential Oils of Thymus Algeriensis, Eucalyptus Globulus and Rosmarinus Officinalis from Morocco. J. Sci. Food Agric..

[44] Almas I., Innocent E., Machumi F., Kisinza W. (2021). Chemical Composition of Essential Oils from Eucalyptus Globulus and Eucalyptus Maculata Grown in Tanzania. Sci. Afr..

[45] Harkat-Madouri L., Asma B., Madani K., Said Z.B.-O.S., Rigou P., Grenier D., Allalou H., Remini H., Adjaoud A., Boulekbache-Makhlouf L. (2015). Chemical Composition, Antibacterial and Antioxidant Activities of Essential Oil of Eucalyptus Globulus from Algeria. Ind. Crops Prod..

[46] Luís Â., Duarte A., Gominho J., Domingues F., Duarte A.P. (2016). Chemical Composition, Antioxidant, Antibacterial and Anti-Quorum Sensing Activities of Eucalyptus Globulus and Eucalyptus Radiata Essential Oils. Ind. Crops Prod..

[47] Pino J.A., Moncayo-Molina L., Spengler I., Pérez J.C. (2021). Chemical Composition and Antibacterial Activity of the Leaf Essential Oil of Eucalyptus Globulus Labill. from Two Highs of the Canton Cañar, Ecuador. Rev. CENIC Cienc. Quím..

[48] Alaba F.B.J., Avestruz P.R.S.D., Cordero R.R.A., Erum E.L., Ng M.R.S., Sumampong M.M.Q., Abiso-Padilla J. (2022). Essential Oils of Allium Sativum and Eucalyptus Globulus as Antagonists for Sars-Cov−2 Infection: A Review. Int. J. Res. Publ. Rev..

[49] Faezeh Taghizadeh S., Panahi A., Esmaeilzadeh Kashi M., Kretschmer N., Asili J., Ahmad Emami S., Azizi M., Shakeri A. (2022). Structural Diversity of Complex Phloroglucinol Derivatives from Eucalyptus Species. Chem. Biodivers..

[50] do Nascimento K.S., Gasparotto Sattler J.A., Lauer Macedo L.F., Serna González C.V., Pereira de Melo I.L., da Silva Araújo E., Granato D., Sattler A., de Almeida-Muradian L.B. (2018). Phenolic Compounds, Antioxidant Capacity and Physicochemical Properties of Brazilian Apis Mellifera Honeys. LWT.

[51] Gül A., Pehlivan T. (2018). Antioxidant Activities of Some Monofloral Honey Types Produced across Turkey. Saudi J. Biol. Sci..

[52] El-Haskoury R., Kriaa W., Lyoussi B., Makni M. (2018). Ceratonia Siliqua Honeys from Morocco: Physicochemical Properties, Mineral Contents, and Antioxidant Activities. J. Food Drug Anal..

[53] El Menyiy N., Akdad M., Elamine Y., Lyoussi B. (2020). Microbiological Quality, Physicochemical Properties, and Antioxidant Capacity of Honey Samples Commercialized in the Moroccan Errachidia Region. J. Food Qual..

[54] Safer A.M., Al-Nughamish A.J. (1999). Hepatotoxicity Induced by the Anti-Oxidant Food Additive, Butylated Hydroxytoluene (BHT), in Rats: An Electron Microscopical Study. Histol. Histopathol..

[55] Aazza S., Lyoussi B., Megías C., Cortés-Giraldo I., Vioque J., Figueiredo A.C., Miguel M.G. (2014). Anti-Oxidant, Anti-Inflammatory and Anti-Proliferative Activities of Moroccan Commercial Essential Oils. Nat. Prod. Commun..

[56] Göger G., Karaca N., Altinbaşak B.B., Demirci B., Demirci F. (2020). In Vitro Antimicrobial, Antioxidant and Anti-Inflammatory Evaluation of Eucalyptus Globulus Essential Oil. Nat. Volatiles Essent. Oils.

[57] Nile S.H., Keum Y.S. (2018). Chemical Composition, Antioxidant, Anti-Inflammatory and Antitumor Activities of Eucalyptus Globulus Labill. Indian J. Exp. Biol..

[58] Vigo E., Cepeda A., Perez-Fernandez R., Gualillo O. (2004). In-Vitro Anti-Inflammatory Effect of Eucalyptus Globulus and Thymus Vulgaris: Nitric Oxide Inhibition in J774 A. 1 Murine Macrophages. J. Pharm. Pharmacol..

[59] Mirke N.B., Shelke P.S., Malavdkar P.R., Jagtap P.N. (2020). In Vitro Protein Denaturation Inhibition Assay of Eucalyptus Globulus and Glycine Max for Potential Anti-Inflammatory Activity. Innov. Pharm. Pharmacother..

[60] Sharma A.D., Farmaha M., Kaur I., Singh N. (2021). Phytochemical Analysis Using GC-FID, FPLC Fingerprinting, Antioxidant, Antimicrobial, Anti-Inflammatory Activities Analysis of Traditionally Used Eucalyptus Globulus Essential Oil. Drug Anal. Res..

[61] Alzubier A.A., Okechukwu P.N. (2011). Investigation of Anti-Inflammatory, Antipyretic and Analgesic Effect of Yemeni Sidr Honey. World Acad. Sci. Eng. Technol..

[62] Silva J., Abebe W., Sousa S.M., Duarte V.G., Machado M.I.L., Matos F.J.A. (2003). Analgesic and Anti-Inflammatory Effects of Essential Oils of Eucalyptus. J. Ethnopharmacol..

[63] Choi I.-Y., Lim J.H., Hwang S., Lee J.-C., Cho G.-S., Kim W.-K. (2010). Anti-Ischemic and Anti-Inflammatory Activity of (S)-Cis-Verbenol. Free Radic. Res..

[64] Li X.-J., Yang Y.-J., Li Y.-S., Zang W.K., Tang H.-B. (2016). A-Pinene, Linalool, and 1-Octanol Contribute to the Topical Anti-Inflammatory and Analgesic Activities of Frankincense by Inhibiting COX−2. J. Ethnopharmacol..

[65] Sugimoto K., Nakagawa K., Hayashi S., Amakura Y., Yoshimura M., Yoshida T., Yamaji R., Nakano Y., Inui H. (2009). Hydrolyzable Tannins as Antioxidants in the Leaf Extract of Eucalyptus Globulus Possessing Tyrosinase and Hyaluronidase Inhibitory Activities. Food Sci. Technol. Res..

[66] Moreira P., Sousa F.J., Matos P., Brites G.S., Gonçalves M.J., Cavaleiro C., Figueirinha A., Salgueiro L., Batista M.T., Branco P.C. (2022). Chemical Composition and Effect against Skin Alterations of Bioactive Extracts Obtained by the Hydrodistillation of Eucalyptus Globulus Leaves. Pharmaceutics.

[67] Bhatt D., Sachan A.K., Jain S., Barik R. (2011). Studies on Inhibitory Effect of Eucalyptus Oil on Sebaceous Glands for the Management of Acne. Indian J. Nat. Prod. Resour..

[68] Kubera Sampath Kumar S., Prakash C., Ramesh P., Sukumar N., Balaji J., Palaniswamy N.K. (2021). Study of Wound Dressing Material Coated with Natural Extracts of Calotropis Gigantean, Eucalyptus Globulus and Buds of Syzygium Aromaticum Solution Enhanced with RhEGF (REGEN-DTM 60). J. Nat. Fibers.

[69] Al-Hisnawi A.A., Mustafa J.M., Yasser Y.K. (2019). The Antimicrobial Activity Synergism between Eucalyptus Honey, Pomegranate, Date and Antibiotics on Escherichia Coli Causing Diarrhea in Children. AIP Conf. Proc..

[70] Bachir R.G., Benali M. (2012). Antibacterial Activity of the Essential Oils from the Leaves of Eucalyptus Globulus against Escherichia Coli and Staphylococcus Aureus. Asian Pac. J. Trop. Biomed..

[71] Djenane D., Yangüela J., Amrouche T., Boubrit S., Boussad N., Roncalés P. (2011). Chemical Composition and Antimicrobial Effects of Essential Oils of Eucalyptus Globulus, Myrtus Communis and Satureja Hortensis against Escherichia Coli O157:H7 and Staphylococcus Aureus in Minced Beef. Food Sci. Technol. Int. Cienc. Tecnol. Los Aliment. Int..

[72] Damjanović-Vratnica B., Đakov T., Šuković D., Damjanović J. (2011). Antimicrobial Effect of Essential Oil Isolated from Eucalyptus Globulus Labill. from Montenegro. Czech J. Food Sci..

